# Supplementary material for: Collapsing the list of myocardial infarction-related differentially expressed genes into a diagnostic signature
Source: J Transl Med. 2020 Jun 9;18:231. doi: 10.1186/s12967-020-02400-1 (PMC7285786; doi:10.1186/s12967-020-02400-1)
Supplement: Supplementary file 1 — Additional file 1: Table S1. The differentially expressed genes identified in PBMC of MI patients when compared to healthy individuals. Figure S1. Correlation matrix plot of the expression levels of gene MIR21 and target genes of this miRNA. Figure S2. Correlation matrix plot of the expression levels of gene MIR223 and target gene MAFB of this miRNA. Figure S3. Correlation matrix plot of the expression levels of differentially expressed genes from “Neutrophil degranulation” Reactome set. Figure S4. Correlation matrix plot of the expression levels of differentially expressed genes from “Cytokine Signaling in Immune system” Reactome set and of MIR21 and target genes of this miRNA. Figure S5. Correlation matrix plot of the expression levels of differentially expressed genes from “Immunoregulatory interactions between a Lymphoid and a non-Lymphoid cell” Reactome set. [file 12967_2020_2400_MOESM1_ESM.docx]

**Additional file 1**:

**Tables**

Table S1. The differentially expressed genes identified in PBMC of MI patients when compared to healthy individuals.

| **Genes** | **Chr** | **Band** | **logFC** | **p-value** | **p.adj** |
| --- | --- | --- | --- | --- | --- |
| *HLX* | 1 | q41 | 0.53 | 3.95E-04 | 7.52E-02 |
| *FCRL3* | 1 | q23.1 | -0.67 | 2.17E-03 | 1.23E-01 |
| *FCGR1B* | 1 | p11.2 | 0.80 | 3.68E-03 | 1.46E-01 |
| *FCGR1A* | 1 | q21.2 | 0.81 | 4.71E-03 | 1.57E-01 |
| *S100A12* | 1 | q21.3 | 0.70 | 4.78E-03 | 1.57E-01 |
| *LAX1* | 1 | q32.1 | -0.51 | 4.96E-03 | 1.59E-01 |
| *SORT1* | 1 | p13.3 | 0.53 | 5.63E-03 | 1.65E-01 |
| *PADI2* | 1 | p36.13 | 0.61 | 5.64E-03 | 1.65E-01 |
| *TGFBR3* | 1 | p22.1 | -0.56 | 7.32E-03 | 1.79E-01 |
| *CR1* | 1 | q32.2 | 0.58 | 3.57E-02 | 2.87E-01 |
| *MERTK* | 2 | q13 | 0.77 | 1.19E-04 | 5.25E-02 |
| *SLC11A1* | 2 | q35 | 0.59 | 2.38E-04 | 6.68E-02 |
| *DYSF* | 2 | p13.2 | 0.80 | 1.33E-02 | 2.10E-01 |
| *CYP1B1* | 2 | p22.2 | 0.50 | 2.24E-02 | 2.47E-01 |
| *BCL6* | 3 | q27.3 | 0.80 | 3.38E-03 | 1.41E-01 |
| *CCR1* | 3 | p21.31 | 0.55 | 1.97E-02 | 2.40E-01 |
| *GASK1B* | 4 | q32.1 | 0.95 | 7.61E-04 | 9.24E-02 |
| *TLR2* | 4 | q31.3 | 0.70 | 5.09E-03 | 1.60E-01 |
| *BST1* | 4 | p15.32 | 0.54 | 5.13E-03 | 1.60E-01 |
| *WDFY3* | 4 | q21.23 | 0.52 | 5.77E-03 | 1.66E-01 |
| *FGFBP2* | 4 | p15.32 | -0.82 | 5.89E-03 | 1.67E-01 |
| *ACSL1* | 4 | q35.1 | 0.77 | 1.39E-02 | 2.14E-01 |
| *HRH2* | 5 | q35.2 | 0.58 | 1.18E-04 | 5.25E-02 |
| *CD14* | 5 | q31.3 | 0.57 | 5.09E-03 | 1.60E-01 |
| *ENPP4* | 6 | p21.1 | -0.50 | 6.10E-04 | 8.89E-02 |
| *VNN3* | 6 | q23.2 | 0.74 | 1.17E-02 | 2.03E-01 |
| *HLA-DQB1* | 6 | p21.32 | 0.79 | 2.24E-02 | 2.47E-01 |
| *SGK1* | 6 | q23.2 | 0.54 | 3.91E-02 | 2.97E-01 |
| *TRGV8* | 7 | p14.1 | -0.70 | 1.48E-03 | 1.09E-01 |
| *TRGV2* | 7 | p14.1 | -0.76 | 2.82E-02 | 2.67E-01 |
| *TRBV25-1* | 7 | q34 | -0.80 | 4.43E-02 | 3.10E-01 |
| *MYBL1* | 8 | q13.1 | -0.80 | 2.15E-03 | 1.22E-01 |
| *NRG1* | 8 | p12 | 0.63 | 4.93E-02 | 3.23E-01 |
| *S1PR3* | 9 | q22.1 | 0.77 | 3.06E-04 | 7.07E-02 |
| *CEP78* | 9 | q21.2 | -0.59 | 7.94E-04 | 9.35E-02 |
| *PRF1* | 10 | q22.1 | -0.58 | 3.99E-04 | 7.52E-02 |
| *LIPN* | 10 | q23.31 | 0.61 | 1.66E-02 | 2.27E-01 |
| *PDGFD* | 11 | q22.3 | -0.52 | 1.29E-04 | 5.46E-02 |
| *SYTL2* | 11 | q14.1 | -0.55 | 6.48E-03 | 1.73E-01 |
| *KLRB1* | 12 | p13.31 | -0.64 | 3.82E-05 | 4.51E-02 |
| *KLRD1* | 12 | p13.2 | -0.77 | 3.78E-04 | 7.50E-02 |
| *C12orf75* | 12 | q23.3 | -0.51 | 1.74E-03 | 1.14E-01 |
| *KLRF1* | 12 | p13.31 | -0.65 | 1.90E-03 | 1.18E-01 |
| *DRAM1* | 12 | q23.2 | 0.50 | 5.70E-03 | 1.65E-01 |
| *KLRC3* | 12 | p13.2 | -0.98 | 7.43E-03 | 1.80E-01 |
| *GLT1D1* | 12 | q24.33 | 0.55 | 8.93E-03 | 1.89E-01 |
| *KLRC2* | 12 | p13.2 | -0.74 | 1.04E-02 | 1.98E-01 |
| *KLRC1* | 12 | p13.2 | -0.69 | 1.09E-02 | 2.00E-01 |
| *PLBD1* | 12 | p13.1 | 0.57 | 1.24E-02 | 2.06E-01 |
| *IFNG* | 12 | q15 | -0.72 | 1.26E-02 | 2.07E-01 |
| *CD163* | 12 | p13.31 | 0.63 | 1.54E-02 | 2.22E-01 |
| *C3AR1* | 12 | p13.31 | 0.60 | 1.88E-02 | 2.36E-01 |
| *CLEC4D* | 12 | p13.31 | 0.81 | 3.75E-02 | 2.93E-01 |
| *LIN7A* | 12 | q21.31 | 0.53 | 4.24E-02 | 3.05E-01 |
| *CLEC6A* | 12 | p13.31 | 0.50 | 4.82E-02 | 3.20E-01 |
| *FLT3* | 13 | q12.2 | 0.55 | 1.05E-03 | 1.00E-01 |
| *GZMB* | 14 | q12 | -0.74 | 2.40E-03 | 1.27E-01 |
| *GZMH* | 14 | q12 | -0.86 | 7.00E-03 | 1.76E-01 |
| *TRAJ19* | 14 | q11.2 | -0.62 | 9.05E-03 | 1.90E-01 |
| *PYGL* | 14 | q22.1 | 0.59 | 9.17E-03 | 1.91E-01 |
| *TRAV30* | 14 | q11.2 | -0.63 | 4.47E-02 | 3.11E-01 |
| *THBS1* | 15 | q14 | 1.15 | 2.12E-03 | 1.21E-01 |
| *AQP9* | 15 | q21.3 | 0.78 | 4.93E-02 | 3.22E-01 |
| *ADGRG1* | 16 | q21 | -0.57 | 1.02E-03 | 1.00E-01 |
| *ADAP2* | 17 | q11.2 | 0.64 | 8.70E-05 | 4.95E-02 |
| *TBX21* | 17 | q21.32 | -0.52 | 2.52E-03 | 1.29E-01 |
| *MIR21* | 17 | q23.1 | 1.51 | 5.45E-03 | 1.64E-01 |
| *ASGR2* | 17 | p13.1 | 0.51 | 7.20E-03 | 1.78E-01 |
| *GRN* | 17 | q21.31 | 0.51 | 1.41E-02 | 2.14E-01 |
| *SOCS3* | 17 | q25.3 | 0.66 | 1.67E-02 | 2.27E-01 |
| *NOG* | 17 | q22 | -0.55 | 1.88E-02 | 2.36E-01 |
| *AC139677.2* | 17 | 17q21.32 | 0.65 | 2.79E-02 | 2.66E-01 |
| *CCDC144A* | 17 | p11.2 | -0.59 | 3.83E-02 | 2.95E-01 |
| *NKG7* | 19 | q13.41 | -0.61 | 4.74E-04 | 7.96E-02 |
| *FPR1* | 19 | q13.41 | 0.66 | 6.74E-03 | 1.74E-01 |
| *KIR2DL1* | 19 | 19q13.42 | -0.59 | 2.70E-02 | 2.63E-01 |
| *FPR2* | 19 | q13.41 | 0.65 | 4.06E-02 | 3.01E-01 |
| *KIR2DL3* | 19 | 19q13.42 | -0.60 | 4.11E-02 | 3.02E-01 |
| *MAFB* | 20 | q12 | 0.65 | 2.16E-02 | 2.45E-01 |
| *MIR223* | X | q12 | 0.63 | 9.43E-03 | 1.93E-01 |
| *TLR8* | X | p22.2 | 0.51 | 1.87E-02 | 2.36E-01 |
| *VSIG4* | X | q12 | 0.55 | 3.93E-02 | 2.98E-01 |

**Figures**


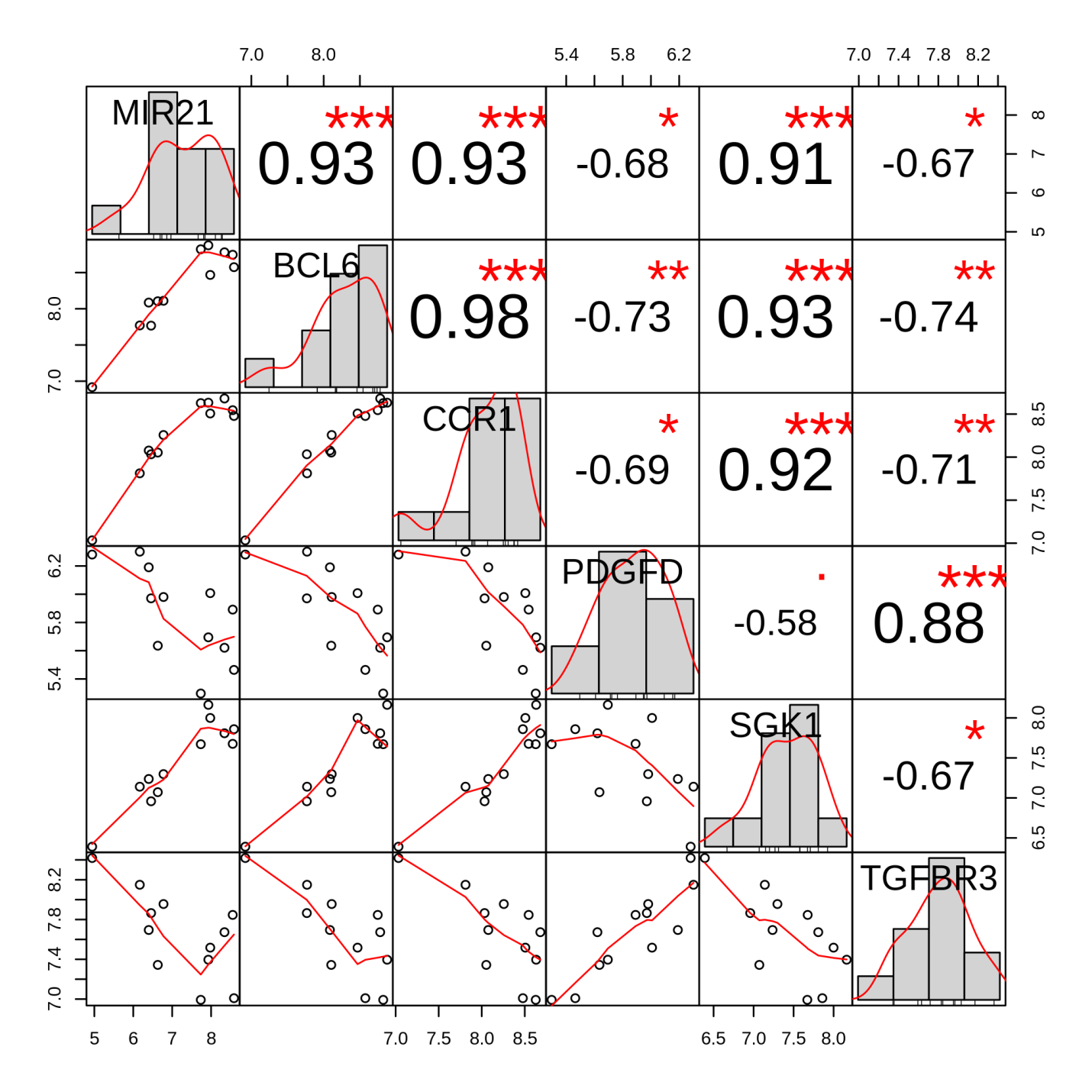


Figure S1. Correlation matrix plot of the expression levels of gene *MIR21* and target genes of this miRNA. The lower triangular matrix is composed by the scatter plots for expression levels of corresponding genes with a fitted smooth line; the upper triangular matrix shows the Spearman correlation coefficients plus significance level (as stars and squares). The size of numbers indicates the absolute value of correlation coefficient. Each significance level is associated to a symbol: p-values<0.001 (***),<0.01 (**),<0.05 (*),<0.1 (^◼️^). The diagonal panel presents genes and its expression levels’ histograms.


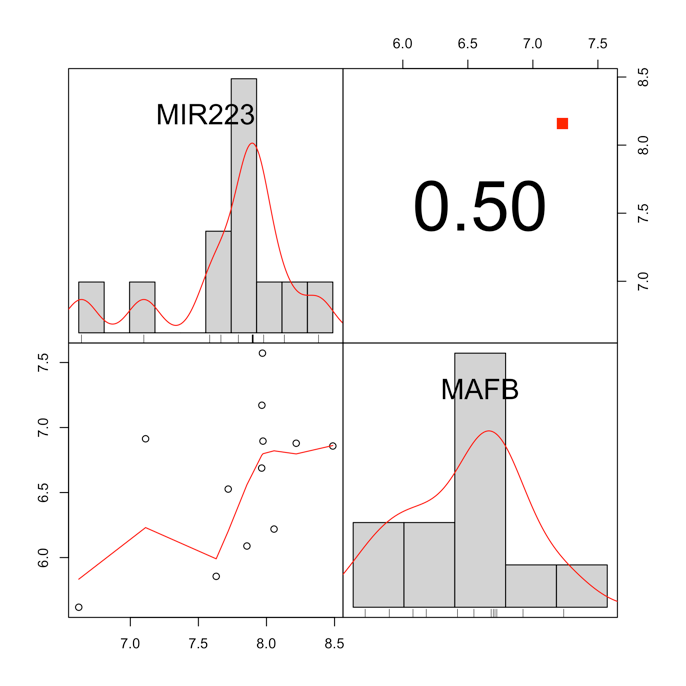


Figure S2. Correlation matrix plot of the expression levels of gene *MIR223* and target gene *MAFB* of this miRNA. The lower triangular is composed by the scatter plot for expression levels of corresponding genes with a fitted smooth line; the upper triangular matrix shows the Spearman correlation coefficient plus significance level as square (^◼️^) indicates for p<0.1. The diagonal panel presents genes and its expression levels’ histograms.


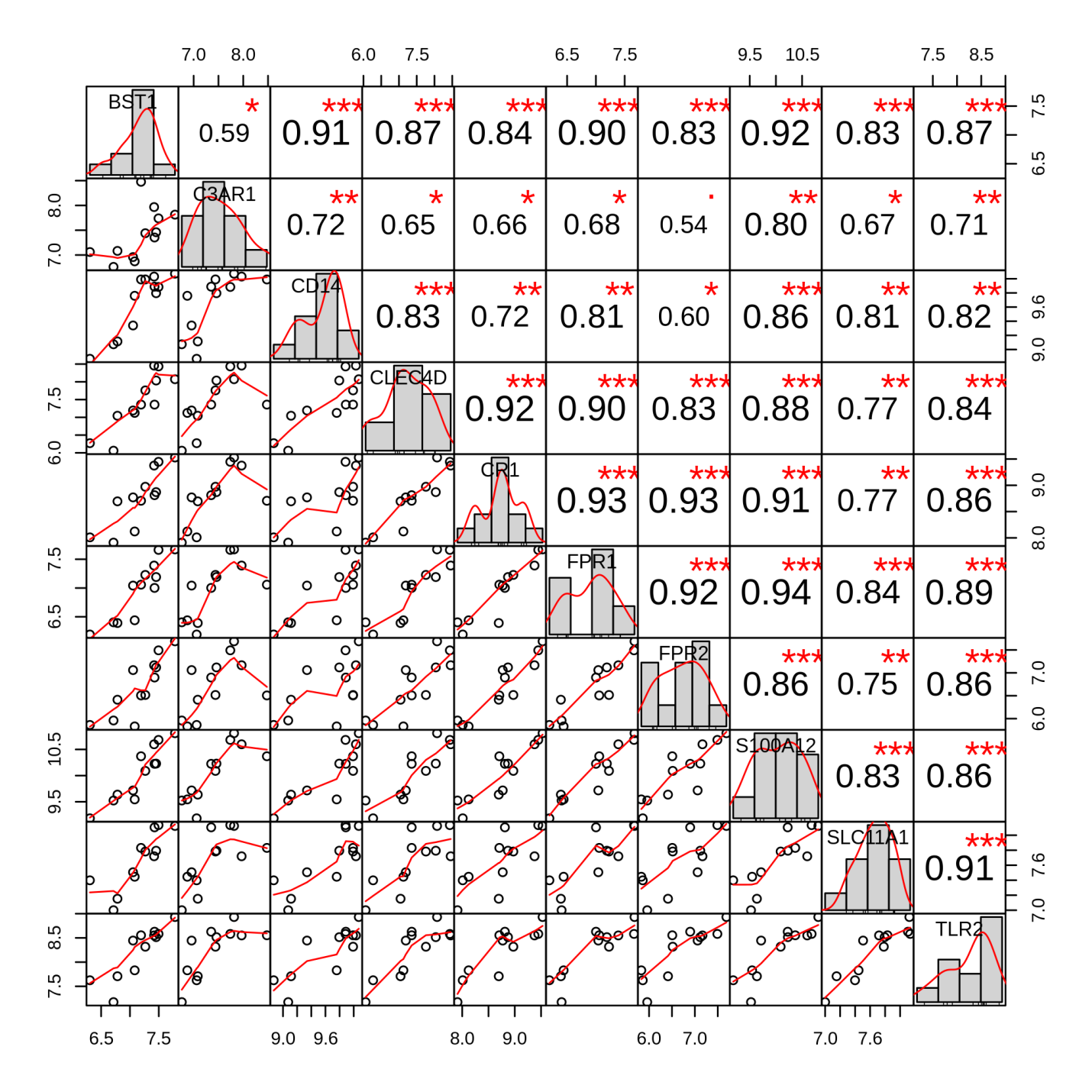


Figure S3. Correlation matrix plot of the expression levels of differentially expressed genes from “Neutrophil degranulation” Reactome set. The lower triangular matrix is composed by the scatter plots for expression levels of corresponding genes with a fitted smooth line; the upper triangular matrix shows the Spearman correlation coefficients plus significance level (as stars and squares). The size of numbers indicates the absolute value of correlation coefficient. Each significance level is associated to a symbol: p-values<0.001 (***),<0.01 (**),<0.05 (*),<0.1 (^◼️^). The diagonal panel presents genes and its expression levels’ histograms.


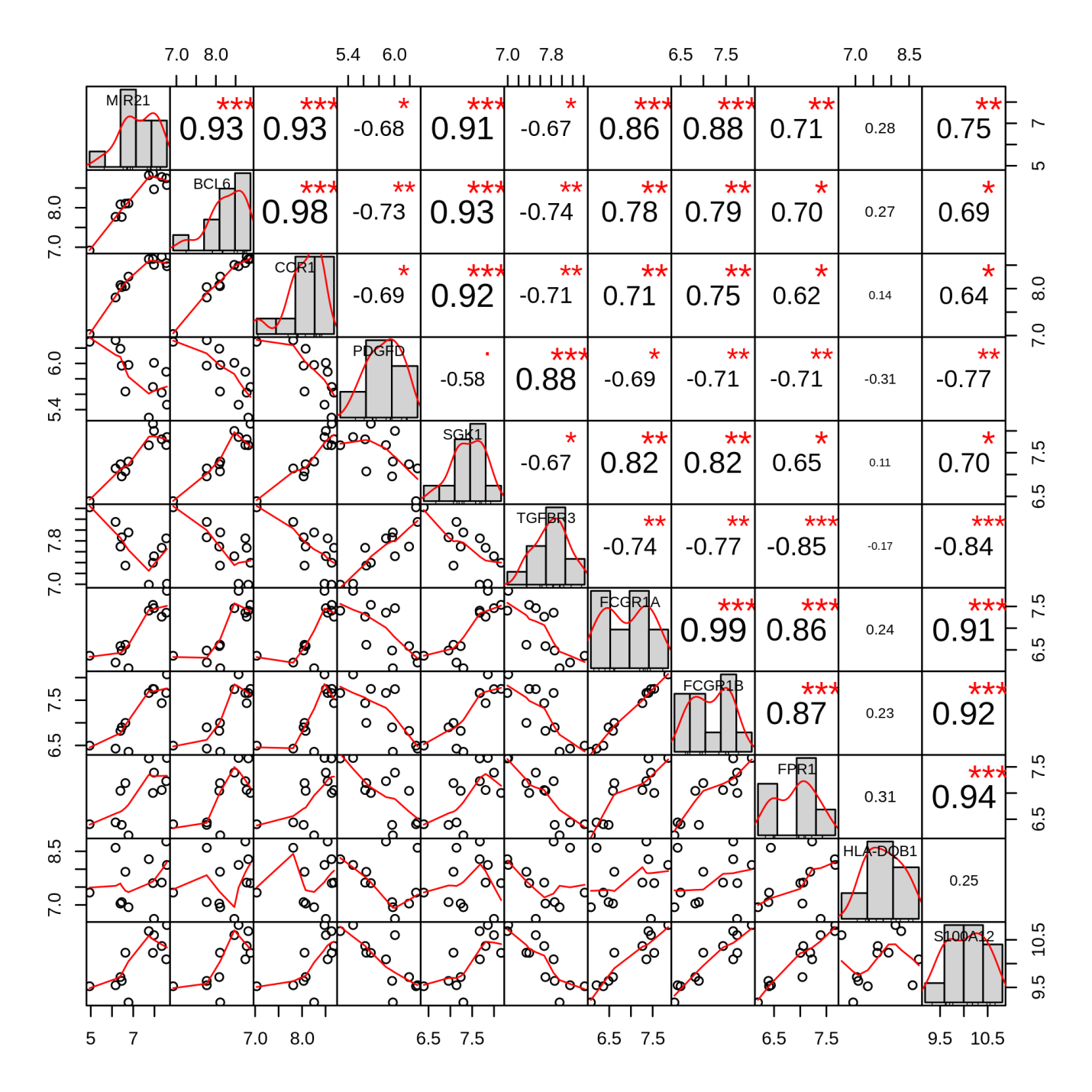


Figure S4. Correlation matrix plot of the expression levels of differentially expressed genes from “Cytokine Signaling in Immune system” Reactome set and of *MIR21* and target genes of this miRNA. The lower triangular matrix is composed by the scatter plots for expression levels of corresponding genes with a fitted smooth line; the upper triangular matrix shows the Spearman correlation coefficients plus significance level (as stars). The size of numbers indicates the absolute value of correlation coefficient. Each significance level is associated to a symbol: p-values<0.001 (***),<0.01 (**),<0.05 (*),<0.1 (^◼️^). The diagonal panel presents genes and its expression levels’ histograms.


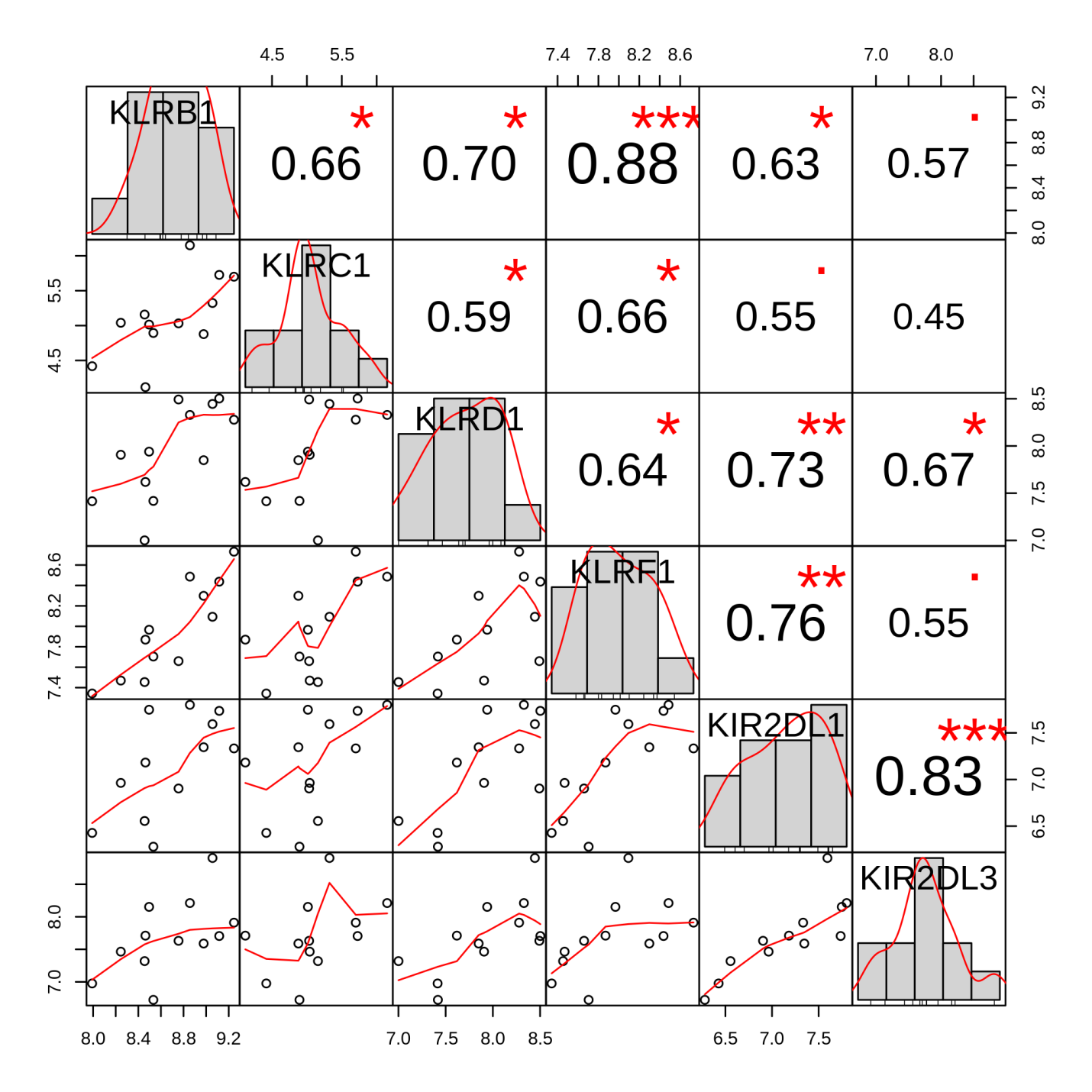


Figure S5. Correlation matrix plot of the expression levels of differentially expressed genes from “Immunoregulatory interactions between a Lymphoid and a non-Lymphoid cell” Reactome set. The lower triangular matrix is composed by the scatter plots for expression levels of corresponding genes with a fitted smooth line; the upper triangular matrix shows the Spearman correlation coefficients plus significance level (as stars and squares). The size of numbers indicates the absolute value of correlation coefficient. Each significance level is associated to a symbol: p-values<0.001 (***),<0.01 (**),<0.05 (*),<0.1 (^◼️^). The diagonal panel presents genes and its expression levels’ histograms.
